# Supplementary material for: Human milk microbiota associated with early colonization of the neonatal gut in Mexican newborns
Source: PeerJ. 2020 May 22;8:e9205. doi: 10.7717/peerj.9205 (PMC7247532; doi:10.7717/peerj.9205)
Supplement: Table S1 [file peerj-08-9205-s001.docx]

| **Table S1: List of 100 barcode adapters sequences for each sequencing primers.** | | | | |
| --- | --- | --- | --- | --- |
| **Name** | **Ion Torrent Linker** | **Golay Barcode** | **Spacer** | **16S rRNA Primer (5'🡪3')** |
| V3-341 F1 | CCATCTCATCCCTGCGTGTCTCCGACTCAG | GATCTGCGATCC | GT | CCTACGGGAGGCAGCAG |
| V3-341 F2 | CCATCTCATCCCTGCGTGTCTCCGACTCAG | CAGCTCATCAGC | GT | CCTACGGGAGGCAGCAG |
| V3-341 F3 | CCATCTCATCCCTGCGTGTCTCCGACTCAG | CAAACAACAGCT | GT | CCTACGGGAGGCAGCAG |
| V3-341 F4 | CCATCTCATCCCTGCGTGTCTCCGACTCAG | GCAACACCATCC | GT | CCTACGGGAGGCAGCAG |
| V3-341 F5 | CCATCTCATCCCTGCGTGTCTCCGACTCAG | GCGATATATCGC | GT | CCTACGGGAGGCAGCAG |
| V3-341 F6 | CCATCTCATCCCTGCGTGTCTCCGACTCAG | CGAGCAATCCTA | GT | CCTACGGGAGGCAGCAG |
| V3-341 F7 | CCATCTCATCCCTGCGTGTCTCCGACTCAG | AGTCGTGCACAT | GT | CCTACGGGAGGCAGCAG |
| V3-341 F8 | CCATCTCATCCCTGCGTGTCTCCGACTCAG | GTATCTGCGCGT | GT | CCTACGGGAGGCAGCAG |
| V3-341 F9 | CCATCTCATCCCTGCGTGTCTCCGACTCAG | CGAGGGAAAGTC | GT | CCTACGGGAGGCAGCAG |
| V3-341 F10 | CCATCTCATCCCTGCGTGTCTCCGACTCAG | CAAATTCGGGAT | GT | CCTACGGGAGGCAGCAG |
| V3-341 F11 | CCATCTCATCCCTGCGTGTCTCCGACTCAG | AGATTGACCAAC | GT | CCTACGGGAGGCAGCAG |
| V3-341 F12 | CCATCTCATCCCTGCGTGTCTCCGACTCAG | AGTTACGAGCTA | GT | CCTACGGGAGGCAGCAG |
| V3-341 F13 | CCATCTCATCCCTGCGTGTCTCCGACTCAG | GCATATGCACTG | GT | CCTACGGGAGGCAGCAG |
| V3-341 F14 | CCATCTCATCCCTGCGTGTCTCCGACTCAG | CAACTCCCGTGA | GT | CCTACGGGAGGCAGCAG |
| V3-341 F15 | CCATCTCATCCCTGCGTGTCTCCGACTCAG | TTGCGTTAGCAG | GT | CCTACGGGAGGCAGCAG |
| V3-341 F16 | CCATCTCATCCCTGCGTGTCTCCGACTCAG | TACGAGCCCTAA | GT | CCTACGGGAGGCAGCAG |
| V3-341 F17 | CCATCTCATCCCTGCGTGTCTCCGACTCAG | CACTACGCTAGA | GT | CCTACGGGAGGCAGCAG |
| V3-341 F18 | CCATCTCATCCCTGCGTGTCTCCGACTCAG | TGCAGTCCTCGA | GT | CCTACGGGAGGCAGCAG |
| V3-341 F19 | CCATCTCATCCCTGCGTGTCTCCGACTCAG | ACCATAGCTCCG | GT | CCTACGGGAGGCAGCAG |
| V3-341 F20 | CCATCTCATCCCTGCGTGTCTCCGACTCAG | TGGACATCTCTT | GT | CCTACGGGAGGCAGCAG |
| V3-341 F21 | CCATCTCATCCCTGCGTGTCTCCGACTCAG | GAACACTTTGGA | GT | CCTACGGGAGGCAGCAG |
| V3-341 F22 | CCATCTCATCCCTGCGTGTCTCCGACTCAG | GAGCCATCTGTA | GT | CCTACGGGAGGCAGCAG |
| V3-341 F23 | CCATCTCATCCCTGCGTGTCTCCGACTCAG | TTGGGTACACGT | GT | CCTACGGGAGGCAGCAG |
| V3-341 F24 | CCATCTCATCCCTGCGTGTCTCCGACTCAG | AAGGCGCTCCTT | GT | CCTACGGGAGGCAGCAG |
| V3-341 F25 | CCATCTCATCCCTGCGTGTCTCCGACTCAG | TAATACGGATCG | GT | CCTACGGGAGGCAGCAG |
| V3-341 F26 | CCATCTCATCCCTGCGTGTCTCCGACTCAG | TCGGAATTAGAC | GT | CCTACGGGAGGCAGCAG |
| V3-341 F27 | CCATCTCATCCCTGCGTGTCTCCGACTCAG | TGTGAATTCGGA | GT | CCTACGGGAGGCAGCAG |
| V3-341 F28 | CCATCTCATCCCTGCGTGTCTCCGACTCAG | CATTCGTGGCGT | GT | CCTACGGGAGGCAGCAG |
| V3-341 F29 | CCATCTCATCCCTGCGTGTCTCCGACTCAG | AACGCACGCTAG | GT | CCTACGGGAGGCAGCAG |
| V3-341 F30 | CCATCTCATCCCTGCGTGTCTCCGACTCAG | ACACTGTTCATG | GT | CCTACGGGAGGCAGCAG |
| V3-341 F31 | CCATCTCATCCCTGCGTGTCTCCGACTCAG | ACCAGACGATGC | GT | CCTACGGGAGGCAGCAG |
| V3-341 F32 | CCATCTCATCCCTGCGTGTCTCCGACTCAG | ACGCTCATGGAT | GT | CCTACGGGAGGCAGCAG |
| V3-341 F33 | CCATCTCATCCCTGCGTGTCTCCGACTCAG | ACTCACGGTATG | GT | CCTACGGGAGGCAGCAG |
| V3-341 F34 | CCATCTCATCCCTGCGTGTCTCCGACTCAG | AGACCGTCAGAC | GT | CCTACGGGAGGCAGCAG |
| V3-341 F35 | CCATCTCATCCCTGCGTGTCTCCGACTCAG | AGCACGAGCCTA | GT | CCTACGGGAGGCAGCAG |
| V3-341 F36 | CCATCTCATCCCTGCGTGTCTCCGACTCAG | ACAGACCACTCA | GT | CCTACGGGAGGCAGCAG |
| V3-341 F37 | CCATCTCATCCCTGCGTGTCTCCGACTCAG | ACCAGCGACTAG | GT | CCTACGGGAGGCAGCAG |
| V3-341 F38 | CCATCTCATCCCTGCGTGTCTCCGACTCAG | ACGGATCGTCAG | GT | CCTACGGGAGGCAGCAG |
| V3-341 F39 | CCATCTCATCCCTGCGTGTCTCCGACTCAG | AGCTTGACAGCT | GT | CCTACGGGAGGCAGCAG |
| V3-341 F40 | CCATCTCATCCCTGCGTGTCTCCGACTCAG | AACTGTGCGTAC | GT | CCTACGGGAGGCAGCAG |
| V3-341 F41 | CCATCTCATCCCTGCGTGTCTCCGACTCAG | ACCGCAGAGTCA | GT | CCTACGGGAGGCAGCAG |
| V3-341 F42 | CCATCTCATCCCTGCGTGTCTCCGACTCAG | ACGGTGAGTGTC | GT | CCTACGGGAGGCAGCAG |
| V3-341 F43 | CCATCTCATCCCTGCGTGTCTCCGACTCAG | ACTCGATTCGAT | GT | CCTACGGGAGGCAGCAG |
| V3-341 F44 | CCATCTCATCCCTGCGTGTCTCCGACTCAG | AGACTGCGTACT | GT | CCTACGGGAGGCAGCAG |
| V3-341 F45 | CCATCTCATCCCTGCGTGTCTCCGACTCAG | AGCAGTCGCGAT | GT | CCTACGGGAGGCAGCAG |
| V3-341 F46 | CCATCTCATCCCTGCGTGTCTCCGACTCAG | AGGACGCACTGT | GT | CCTACGGGAGGCAGCAG |
| V3-341 F47 | CCATCTCATCCCTGCGTGTCTCCGACTCAG | AAGAGATGTCGA | GT | CCTACGGGAGGCAGCAG |
| V3-341 F48 | CCATCTCATCCCTGCGTGTCTCCGACTCAG | ACAGCAGTGGTC | GT | CCTACGGGAGGCAGCAG |
| V3-341 F49 | CCATCTCATCCCTGCGTGTCTCCGACTCAG | ACGTACTCAGTG | GT | CCTACGGGAGGCAGCAG |
| V3-341 F50 | CCATCTCATCCCTGCGTGTCTCCGACTCAG | ACTCGCACAGGA | GT | CCTACGGGAGGCAGCAG |
| V3-341 F51 | CCATCTCATCCCTGCGTGTCTCCGACTCAG | AGAGAGCAAGTG | GT | CCTACGGGAGGCAGCAG |
| V3-341 F52 | CCATCTCATCCCTGCGTGTCTCCGACTCAG | AGCATATGAGAG | GT | CCTACGGGAGGCAGCAG |
| V3-341 F53 | CCATCTCATCCCTGCGTGTCTCCGACTCAG | AGGCTACACGAC | GT | CCTACGGGAGGCAGCAG |
| V3-341 F54 | CCATCTCATCCCTGCGTGTCTCCGACTCAG | AAGCTGCAGTCG | GT | CCTACGGGAGGCAGCAG |
| V3-341 F55 | CCATCTCATCCCTGCGTGTCTCCGACTCAG | ACAGCTAGCTTG | GT | CCTACGGGAGGCAGCAG |
| V3-341 F56 | CCATCTCATCCCTGCGTGTCTCCGACTCAG | ACCTGTCTCTCT | GT | CCTACGGGAGGCAGCAG |
| V3-341 F57 | CCATCTCATCCCTGCGTGTCTCCGACTCAG | ACGTCTGTAGCA | GT | CCTACGGGAGGCAGCAG |
| V3-341 F58 | CCATCTCATCCCTGCGTGTCTCCGACTCAG | AGAGCAAGAGCA | GT | CCTACGGGAGGCAGCAG |
| V3-341 F59 | CCATCTCATCCCTGCGTGTCTCCGACTCAG | AGCCATACTGAC | GT | CCTACGGGAGGCAGCAG |

|  |  |  |  |  |
| --- | --- | --- | --- | --- |
| **Table S1. (continued). List of 100 barcode adapters sequences for each sequencing primers.** | | | | |
| **Name** | **Ion Torrent Linker** | **Golay Barcode** | **Spacer** | **16S rRNA Primer (5'🡪3')** |
| V3-341 F60 | CCATCTCATCCCTGCGTGTCTCCGACTCAG | AGGTGTGATCGC | GT | CCTACGGGAGGCAGCAG |
| V3-341 F61 | CCATCTCATCCCTGCGTGTCTCCGACTCAG | AATCAGTCTCGT | GT | CCTACGGGAGGCAGCAG |
| V3-341 F62 | CCATCTCATCCCTGCGTGTCTCCGACTCAG | ACGACGTCTTAG | GT | CCTACGGGAGGCAGCAG |
| V3-341 F63 | CCATCTCATCCCTGCGTGTCTCCGACTCAG | ACGTGAGAGAAT | GT | CCTACGGGAGGCAGCAG |
| V3-341 F64 | CCATCTCATCCCTGCGTGTCTCCGACTCAG | ACTGACAGCCAT | GT | CCTACGGGAGGCAGCAG |
| V3-341 F65 | CCATCTCATCCCTGCGTGTCTCCGACTCAG | AGCGACTGTGCA | GT | CCTACGGGAGGCAGCAG |
| V3-341 F66 | CCATCTCATCCCTGCGTGTCTCCGACTCAG | AGTACGCTCGAG | GT | CCTACGGGAGGCAGCAG |
| V3-341 F67 | CCATCTCATCCCTGCGTGTCTCCGACTCAG | AATCGTGACTCG | GT | CCTACGGGAGGCAGCAG |
| V3-341 F68 | CCATCTCATCCCTGCGTGTCTCCGACTCAG | ACGAGTGCTATC | GT | CCTACGGGAGGCAGCAG |
| V3-341 F69 | CCATCTCATCCCTGCGTGTCTCCGACTCAG | ACTGATCCTAGT | GT | CCTACGGGAGGCAGCAG |
| V3-341 F70 | CCATCTCATCCCTGCGTGTCTCCGACTCAG | AGAGTCCTGAGC | GT | CCTACGGGAGGCAGCAG |
| V3-341 F71 | CCATCTCATCCCTGCGTGTCTCCGACTCAG | AGCGAGCTATCT | GT | CCTACGGGAGGCAGCAG |
| V3-341 F72 | CCATCTCATCCCTGCGTGTCTCCGACTCAG | AGTACTGCAGGC | GT | CCTACGGGAGGCAGCAG |
| V3-341 F73 | CCATCTCATCCCTGCGTGTCTCCGACTCAG | ACACACTATGGC | GT | CCTACGGGAGGCAGCAG |
| V3-341 F74 | CCATCTCATCCCTGCGTGTCTCCGACTCAG | ACGATGCGACCA | GT | CCTACGGGAGGCAGCAG |
| V3-341 F75 | CCATCTCATCCCTGCGTGTCTCCGACTCAG | ACGTTAGCACAC | GT | CCTACGGGAGGCAGCAG |
| V3-341 F76 | CCATCTCATCCCTGCGTGTCTCCGACTCAG | ACTGTACGCGTA | GT | CCTACGGGAGGCAGCAG |
| V3-341 F77 | CCATCTCATCCCTGCGTGTCTCCGACTCAG | AGATACACGCGC | GT | CCTACGGGAGGCAGCAG |
| V3-341 F78 | CCATCTCATCCCTGCGTGTCTCCGACTCAG | AGCGCTGATGTG | GT | CCTACGGGAGGCAGCAG |
| V3-341 F79 | CCATCTCATCCCTGCGTGTCTCCGACTCAG | ACACATGTCTAC | GT | CCTACGGGAGGCAGCAG |
| V3-341 F80 | CCATCTCATCCCTGCGTGTCTCCGACTCAG | ACATGATCGTTC | GT | CCTACGGGAGGCAGCAG |
| V3-341 F81 | CCATCTCATCCCTGCGTGTCTCCGACTCAG | ACGCAACTGCTA | GT | CCTACGGGAGGCAGCAG |
| V3-341 F82 | CCATCTCATCCCTGCGTGTCTCCGACTCAG | ACTGTCGAAGCT | GT | CCTACGGGAGGCAGCAG |
| V3-341 F83 | CCATCTCATCCCTGCGTGTCTCCGACTCAG | AGCGTAGGTCGT | GT | CCTACGGGAGGCAGCAG |
| V3-341 F84 | CCATCTCATCCCTGCGTGTCTCCGACTCAG | ACACGAGCCACA | GT | CCTACGGGAGGCAGCAG |
| V3-341 F85 | CCATCTCATCCCTGCGTGTCTCCGACTCAG | ACATGTCACGTG | GT | CCTACGGGAGGCAGCAG |
| V3-341 F86 | CCATCTCATCCCTGCGTGTCTCCGACTCAG | ACGCGATACTGG | GT | CCTACGGGAGGCAGCAG |
| V3-341 F87 | CCATCTCATCCCTGCGTGTCTCCGACTCAG | ACTACGTGTGGT | GT | CCTACGGGAGGCAGCAG |
| V3-341 F88 | CCATCTCATCCCTGCGTGTCTCCGACTCAG | ACTGTGACTTCA | GT | CCTACGGGAGGCAGCAG |
| V3-341 F89 | CCATCTCATCCCTGCGTGTCTCCGACTCAG | AGATCTCTGCAT | GT | CCTACGGGAGGCAGCAG |
| V3-341 F90 | CCATCTCATCCCTGCGTGTCTCCGACTCAG | AGCTATCCACGA | GT | CCTACGGGAGGCAGCAG |
| V3-341 F91 | CCATCTCATCCCTGCGTGTCTCCGACTCAG | AGTCCATAGCTG | GT | CCTACGGGAGGCAGCAG |
| V3-341 F92 | CCATCTCATCCCTGCGTGTCTCCGACTCAG | ACACGGTGTCTA | GT | CCTACGGGAGGCAGCAG |
| V3-341 F93 | CCATCTCATCCCTGCGTGTCTCCGACTCAG | ACATTCAGCGCA | GT | CCTACGGGAGGCAGCAG |
| V3-341 F94 | CCATCTCATCCCTGCGTGTCTCCGACTCAG | ACTTGTAGCAGC | GT | CCTACGGGAGGCAGCAG |
| V3-341 F95 | CCATCTCATCCCTGCGTGTCTCCGACTCAG | AGATGTTCTGCT | GT | CCTACGGGAGGCAGCAG |
| V3-341 F96 | CCATCTCATCCCTGCGTGTCTCCGACTCAG | AGCTCCATACAG | GT | CCTACGGGAGGCAGCAG |
| V3-341 F97 | CCATCTCATCCCTGCGTGTCTCCGACTCAG | ACGCTATCTGGA | GT | CCTACGGGAGGCAGCAG |
| V3-341 F98 | CCATCTCATCCCTGCGTGTCTCCGACTCAG | ACTATTGTCACG | GT | CCTACGGGAGGCAGCAG |
| V3-341 F99 | CCATCTCATCCCTGCGTGTCTCCGACTCAG | AGAACACGTCTC | GT | CCTACGGGAGGCAGCAG |
| V3-341 F100 | CCATCTCATCCCTGCGTGTCTCCGACTCAG | AGTGAGAGAAGC | GT | CCTACGGGAGGCAGCAG |
| V3-518 R | CCTCTCTATGGGCAGTCGGTGAT | NOT APPLICABLE | CC | ATTACCGCGGCTGCTGG |
