# Supplementary material for: Human milk microbiota associated with early colonization of the neonatal gut in Mexican newborns
Source: PeerJ. 2020 May 22;8:e9205. doi: 10.7717/peerj.9205 (PMC7247532; doi:10.7717/peerj.9205)
Supplement: Table S2 [file peerj-08-9205-s002.docx]

| **Table S2.** **Sequencing summary.** *** | | |
| --- | --- | --- |
| **Parameter** | **Human milk (n=67)** | **Neonatal stool (n=67)** |
| Number of reads  Mean of reads per sample  Lowest sample reads  Highest sample reads | 4’240,314  63,288  5,416  313,468 | 5’335,223  79,630  10,919  342,510 |
| Mean length for sequences* | 167.16 bases ± 48.35 | 170.94 bases ± 44.14 |
| Total of raw read sequences: 9’575,537; Mean lengths: 169.3 ± 46.1.  **Summary of sequencing after trimming at 200 nt.* | | |
