# Supplementary material for: Human milk microbiota associated with early colonization of the neonatal gut in Mexican newborns
Source: PeerJ. 2020 May 22;8:e9205. doi: 10.7717/peerj.9205 (PMC7247532; doi:10.7717/peerj.9205)
Supplement: Table S3 [file peerj-08-9205-s003.docx]

| **Table S3.** **Relative abundance of bacterial families in human milk-neonatal stool pairs.** | | | | | | | | | | |
| --- | --- | --- | --- | --- | --- | --- | --- | --- | --- | --- |
|  | | **Human milk** | | | | | **Neonatal stool** | | | |
| **Phylum** | **Family** | | **n** | **% ± SD** | **CV** | **Range**  **Min-Max** | **n** | **% ± SD** | **CV** | **Range**  **Min-Max** |
| Actinobacteria | Propionibacteriaceae* | | 91.0 | 7.33 ± 7.7 | 1.1 | 0.0-30.8 | 37.3 | 6.30 ± 13.2 | 2.1 | 0.0-63.4 |
|  | Bifidobacteriaceae* | | 28.4 | 0.73 ± 1.6 | 2.2 | 0.0-11.1 | 70.2 | 9.49 ± 20.2 | 2.1 | 0.0-87.5 |
| Bacteroidetes | Weeksellaceae | | 22.4 | 2.13 ± 9.8 | 4.6 | 0.0-74.8 | 6.0 | 1.23 ± 9.3 | 7.6 | 0.0-85.2 |
| Firmicutes | Staphylococcaceae* | | 82.1 | 15.10 ± 25.4 | 1.7 | 0.2-95.6 | 28.4 | 3.80 ± 13.9 | 3.7 | 0.0-84.7 |
|  | Streptococcaceae | | 58.2 | 3.73 ± 10.4 | 2.8 | 0.0-79.0 | 38.8 | 2.72 ± 7.9 | 2.9 | 0.0-54.7 |
|  | Lachnospiraceae | | 61.2 | 2.03 ± 6.1 | 3.0 | 0.0-49.3 | 41.8 | 2.35 ± 4.2 | 1.8 | 0.0-18.4 |
|  | Ruminococcaceae | | 44.8 | 1.14 ± 3.5 | 3.1 | 0.0-28.3 | 3.0 | 1.46 ± 3.0 | 2.1 | 0.0-15.4 |
|  | Clostridiaceae* | | 31.3 | 0.64 ± 1.0 | 1.6 | 0.0- 4.3 | 61.2 | 15.5 ± 29.9 | 7.9 | 0.1-99.6 |
| Proteobacteria | Sphingomonadaceae* | | 88.1 | 13.2 ± 14.4 | 1.1 | 0.0-51.2 | 35.8 | 2.33 ± 4.0 | 1.7 | 0.0-15.4 |
|  | Pseudomonadaceae* | | 71.6 | 9.64 ± 18.6 | 1.9 | 0.1-73.6 | 77.6 | 24.5 ± 30.5 | 1.2 | 0.0-95.9 |
|  | Rhodobacteraceae* | | 61.2 | 9.16 ± 20.7 | 2.3 | 0.0-82.2 | 28.4 | 0.42 ± 0.84 | 2.0 | 0.0-3.9 |
|  | Bradyrhizobiaceae* | | 68.7 | 6.74 ± 8.7 | 1.3 | 0.0-30.0 | 31.3 | 2.05 ± 5.2 | 2.5 | 0.0-32.0 |
|  | Phyllobacteriaceae* | | 59.7 | 3.35 ± 10.1 | 3.0 | 0.0-73.0 | 16.4 | 0.37 ± 1.1 | 3.0 | 0.0-7.4 |
|  | Enterobacteriaceae* | | 44.8 | 1.61 ± 3.7 | 2.3 | 0.0-20.2 | 70.2 | 4.05 ± 9.1 | 2.2 | 0.0-13.7 |
| n, % of number of samples where taxa has a frequency > 0.5; %, percentage of relative abundance of each taxon; SD, standard deviation; CV, coefficient of variation; Range, denotes the minimum and maximum values in the relative abundance for each taxon.  *Comparison between human milk and neonatal stool using nonparametric t-test, *p*<0.005. Data were calculated using SPSS v23. | | | | | | | | | | |
