# Supplementary material for: Human milk microbiota associated with early colonization of the neonatal gut in Mexican newborns
Source: PeerJ. 2020 May 22;8:e9205. doi: 10.7717/peerj.9205 (PMC7247532; doi:10.7717/peerj.9205)
Supplement: Table S4 [file peerj-08-9205-s004.docx]

| **Table S4.** **Linear discriminant analysis (LDA) effect size (LEfSe) analysis per groups.** | | | | |
| --- | --- | --- | --- | --- |
| **Group** | **Taxa** | **LDA score** | ***p*-value** | ***q*-value*** |
| Human milk | *g*_*Staphylococcus* | 4.8401 | 7.80E-11 | 2.06E-08 |
|  | *g_Kaistobacter* | 4.6520 | 2.70E-11 | 1.27E-08 |
|  | *g_Paracoccus* | 4.6141 | 1.39-09 | 1.84E-07 |
|  | *g_Pseudomonas* | 4.5569 | 1.30E-11 | 1.27E-08 |
|  | *g_Bradyrhizobium* | 4.2760 | 1.05E-09 | 1.58E-07 |
|  | f_Phyllobacteriaceae | 4.1827 | 2.83E-10 | 4.99E-08 |
|  | *g_Methylobacterium* | 4.0943 | 2.53E-10 | 4.99E-08 |
|  | *g_Acinetobacter* | 3.9678 | 3.74E-07 | 3.60E-05 |
|  | *g_Propionibacterium* | 3.9547 | 5.24E-06 | 3.70E-04 |
|  | *g_Corynebacterium* | 3.7748 | 5.32E-08 | 5.63E-06 |
|  | o_Streptophyta | 3.7489 | 4.97E-07 | 4.04E-05 |
|  | f_Sphingomonadaceae | 3.6330 | 3.60E-11 | 1.27E-08 |
|  | *g_Microbacterium* | 3.5203 | 2.18E-08 | 2.56E-06 |
|  | f_Gemellaceae | 3.5193 | 4.29E-07 | 3.78E-05 |
| Neonatal stool | f_Pseudomonadaceae | 5.0538 | 1.74E-06 | 1.31E-04 |
|  | *g_Bifidobacterium* | 4.5921 | 1.26E-05 | 8.30E-04 |
|  | *g_Clostridium* | 4.5867 | 0.0002 | 8.27E-03 |
|  | *g_Bacteroides* | 4.5855 | 7.75E-05 | 4.44E-03 |
|  | f_Clostridiaceae | 4.3524 | 4.62E-05 | 2.87E-03 |
|  | f_Enterobacteriaceae | 3.9386 | 7.99E-05 | 4.44E-03 |
| The threshold on the logarithmic LDA score was set to 3.5. *p* and *q* values < 0.05 were considered statistically significant. **p*-value was adjusted with Benjamini-Hochberg method and generated FDR value (*q*-value). The name of each taxon level appears in abbreviation before its taxon. “f”, family; “g”, genus. “LDA” Linear discriminant analysis. | | | | |
