# Supplementary material for: Human milk microbiota associated with early colonization of the neonatal gut in Mexican newborns
Source: PeerJ. 2020 May 22;8:e9205. doi: 10.7717/peerj.9205 (PMC7247532; doi:10.7717/peerj.9205)
Supplement: Table S5 [file peerj-08-9205-s005.docx]

| **Table S5. Comparison of alpha diversity indexes between human milk and neonatal stool samples.** | | | | |  |
| --- | --- | --- | --- | --- | --- |
| Index | Human milk | Neonatal stool | *p-*value | *q-*value | Effect Size |
|  | n=46 | n=60 |  |  |  |
| Observed | 408.24 ± 156.21 | 242.63 ± 213.77 | < 0.001 | 0.0106 | 0.861 |
| Chao1 | 700.58 ± 226.14 | 435.81 ± 339.90 | < 0.001 | 0.0106 | 0.894 |
| Shannon | 2.81 ± 0.95 | 2.01 ± 1.16 | < 0.001 | 0.0106 | 0.745 |
| Simpson | 0.77 ± 0.18 | 0.64 ± 0.25 | 0.006 | 0.6360 | 0.584 |
| Values are mean ± SD, *p*-value were calculated by Mann Whitney U-test. *p-*value was corrected by Benjamini-Hochberg method and generated FDR value (*q-*value). *p* < 0.05 and q < 0.05 are considered statistically significant. Effect size was calculated using Hedges’ *g*. | | | | | |
