# Supplementary material for: Human milk microbiota associated with early colonization of the neonatal gut in Mexican newborns
Source: PeerJ. 2020 May 22;8:e9205. doi: 10.7717/peerj.9205 (PMC7247532; doi:10.7717/peerj.9205)
Supplement: Table S6 [file peerj-08-9205-s006.docx]

| **Table S6. Comparison of alpha diversity indexes between delivery modes in human milk and neonatal stool samples.** | | | | | |  |
| --- | --- | --- | --- | --- | --- | --- |
| Group | Index | Vaginal | C-section | *p-*value | *q-*value | Effect Size |
|  |  | n=32 | n=14 |  |  |  |
| Human milk (n=46) | Observed | 408.24 ± 156.21 | 402.05 ± 152.73 | 0.152 | 1.000 | 0.040 |
|  | Chao1 | 700.58 ± 226.14 | 690.92 ± 221.44 | 0.252 | 1.000 | 0.043 |
|  | Shannon | 2.81 ± 0.95 | 2.76 ± 0.94 | 0.127 | 1.000 | 0.053 |
|  | Simpson | 0.75 (± 0.19) | 0.76 ± 0.18 | 0.086 | 1.000 | 0.053 |
|  |  | n=41 | n=19 |  |  |  |
| Neonatal stool (n=60) | Observed | 380.37 (± 245.37) | 659.29 (± 350.28) | 0.003 | 0.180 | 0.071 |
|  | Chao1 | 649.14 (± 397.60) | 1098.72 (± 566.14) | 0.006 | 0.180 | 0.067 |
|  | Shannon | 1.97 (± 1.10) | 2.73 ± 1.48 | 0.046 | 0.920 | 0.086 |
|  | Simpson | 0.64 (± 0.25) | 0.64 ± 0.25 | 0.082 | 1.000 | 0.123 |
| Values are mean ± SD, *p*-value were calculated by Mann Whitney U-test. *p-*value was corrected by Benjamini-Hochberg method and generated FDR value (*q-*value). *p* < 0.05 and q < 0.05 are considered statistically significant. Effect size was calculated using Hedges’ *g*. | | | | | | |
