# Supplementary material for: Human milk microbiota associated with early colonization of the neonatal gut in Mexican newborns
Source: PeerJ. 2020 May 22;8:e9205. doi: 10.7717/peerj.9205 (PMC7247532; doi:10.7717/peerj.9205)
Supplement: Table S7 [file peerj-08-9205-s007.docx]

| **Table S7: Absolute read counts of bacterial taxa shared in human milk and neonatal stool samples.** | | | | | |
| --- | --- | --- | --- | --- | --- |
| **Taxonomy** | | | | **Absolute counts*** | |
| **Phylum** | **Order** | **Family** | **Genus/species** | **Human milk** | **Neonatal stool** |
| Actinobacteria | Bifidobacteriales | Bifidobaceriaceae | *Bifidobacterium* | 3,428 | 198,361 |
|  | Coriobacteriales | Coriobacteriaceae | *Collinsella aerofaciens* | 2,596 | 28,892 |
|  | Actinomycetales | Propionibacteriaceae | *Propionibacterium acnes* | 131,657 | 101,193 |
|  | Actinomycetales | Corynebacteriaceae | *Corynebacterium* | 13,556 | 7,613 |
|  | Actinomycetales | n. i. | n. i. | 17,946 | 11,953 |
| Bacteroidetes | Bacteroidales | Bacteroidaceae | *Bacteroides* | 680 | 228,979 |
| Firmicutes | Bacillales | Staphylococcaceae | *Staphylococcus* | 801,723 | 172,033 |
|  | Lactobacillales | Streptococcaceae | *Streptococcus* | 26,512 | 91,504 |
|  | Lactobacillales | Streptococcaceae | *Lactococcus* | 30,026 | 13,295 |
|  | Lactobacillales | Enterococcaceae | *Enterococcus* | 3,738 | 168,373 |
|  | Clostridiales | Lachnospiraceae | n. i. | 15,703 | 19,118 |
|  | Clostridiales | Clostridiaceae | *Clostridium gasigenes* | 2,045 | 482,696 |
|  | Clostridiales | Clostridiaceae | n. i. | 4,892 | 466,272 |
|  | Clostridiales | Ruminococcaceae | *Faecalibacterium prausnitzii* | 9,471 | 9,462 |
|  | Clostridiales | n. i. | n. i. | 3,071 | 7,649 |
| Proteobacteria | Pseudomonadaceae | Pseudomonadaceae | n. i. | 71,337 | 1’228,144 |
|  | Pseudomonadales | Pseudomonadaceae | *Pseudomonas* | 38,267 | 1,054 |
|  | Pseudomonadales | Moraxellaceae | *Acinetobacter iwoffii* | 1,027 | 2,641 |
|  | Rhizobiales | Bradyrhizobiaceae | n. i. | 107,385 | 19,007 |
|  | Rhizobiales | Phyllobacteriaceae | n. i. | 45,666 | 5,255 |
|  | Rhizobiales | Methylobacteriaceae | *Methylobacterium* | 35,491 | 7,469 |
|  | Sphingomonadales | Sphingomonadaceae | *Kaistobacter* | 183,502 | 33,759 |
|  | Rhodobacterales | Rhodobacteraceae | *Paracoccus marcusii* | 69,274 | 4,451 |
|  | Enterobacteriales | Enterobacteriaceae | n. i. | 5,253 | 53,701 |
|  | Chromatiales | Chromatiaceae | n. i. | 594 | 3,544 |
| n. i., not identified taxa. *absolute counts of taxa shared in at least 50% of all samples in each group (human milk and neonate stool). | | | | | |
