# Supplementary material for: Human milk microbiota associated with early colonization of the neonatal gut in Mexican newborns
Source: PeerJ. 2020 May 22;8:e9205. doi: 10.7717/peerj.9205 (PMC7247532; doi:10.7717/peerj.9205)
Supplement: Table S8 [file peerj-08-9205-s008.docx]

| **Table S8: Predicted metabolic pathways in human milk and neonate stool microbiotas by PICRUSt.** | | | | |
| --- | --- | --- | --- | --- |
| **Group** | **Feature** | **Differences between means** | ***p*-value** | ***q*-value** |
| Energy  metabolism | Galactose metabolism | 1.621 | 7.90 e^-5^ | 5.29 e^-4^ |
|  | Pentose phosphate pathway | 0.582 | 2.96 e^-7^ | 6.48 e^-6^ |
|  | Amino acid related enzymes | 1.121 | 4.24 e^-3^ | 1.40 e^-2^ |
|  | Fatty acids metabolism | -4.895 | 1.52 e^-5^ | 1.46 e^-4^ |
|  | Peroxisome | -8.136 | 9.85 e^-4^ | 4.09 e^-3^ |
|  | PPAR pathway | -7.919 | 2.39 e^-7^ | 5.59 e^-6^ |
|  | Vitamin B6 metabolism | 0.559 | 7.24 e^-6^ | 8.19 e^-5^ |
| Colonization | Bacterial chemiotaxis | 1.401 | 1.40 e^-3^ | 5.45 e^-3^ |
|  | Bacterial secretion system | -0.913 | 9.70 e^-5^ | 6.12 e^-4^ |
| Immune Function | Antigen processing and presentation | 4.696 | 1.11 e^-3^ | 4.54 e^-3^ |
| *p*-values were corrected by Benjamini-Hochberg method and generated the FDR value (*q-*value). *p* < 0.05 and *q* < 0.05 are considered statistically significant. | | | | |
