# Supplementary figures and images for: Human milk microbiota associated with early colonization of the neonatal gut in Mexican newborns
Source: PeerJ. 2020 May 22;8:e9205. doi: 10.7717/peerj.9205 (PMC7247532; doi:10.7717/peerj.9205)

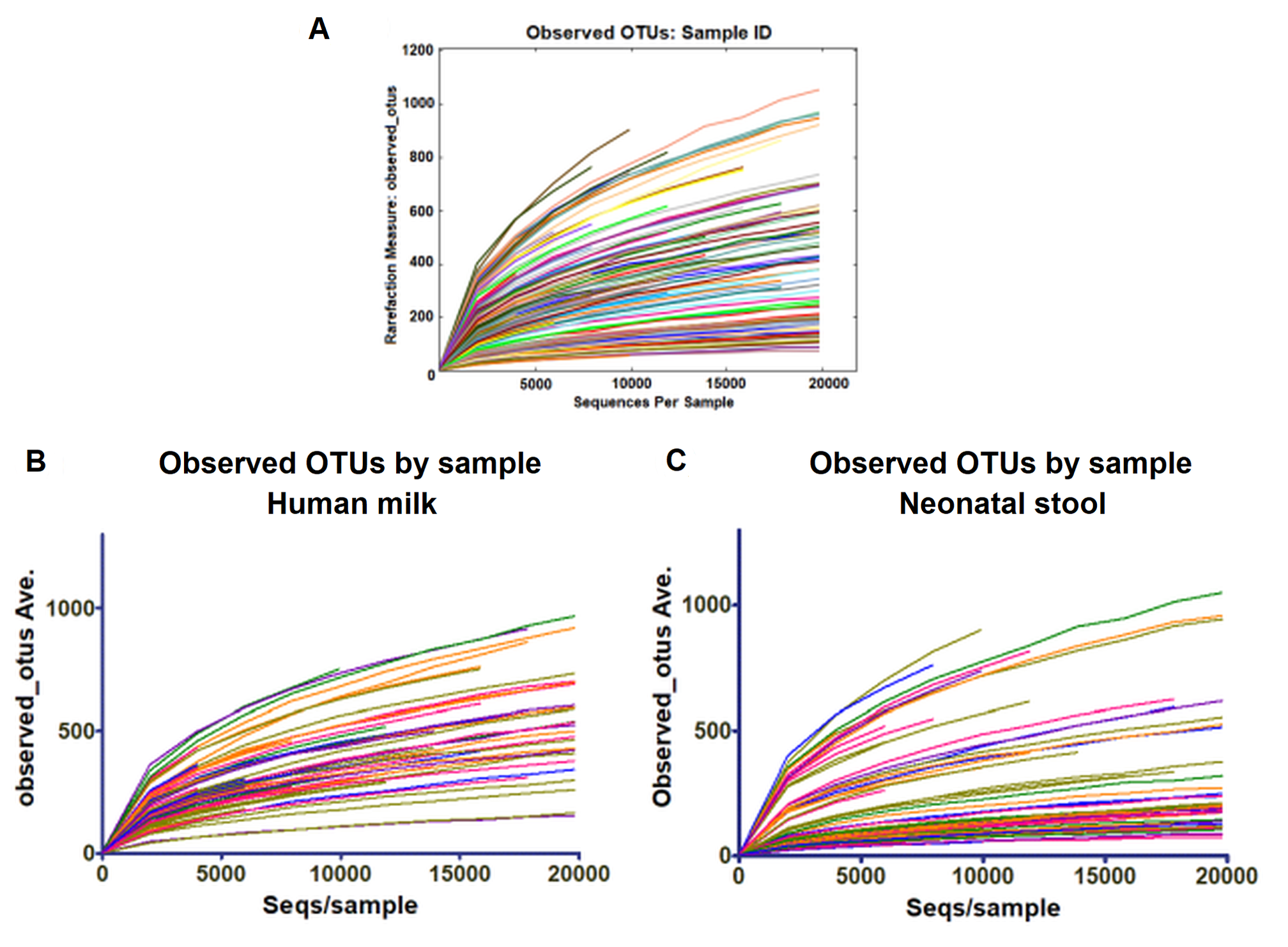

Supplement: Figure S1 — Plots show total samples (A), human milk (B) and neonatal stool (C) groups. [file peerj-08-9205-s009.png]

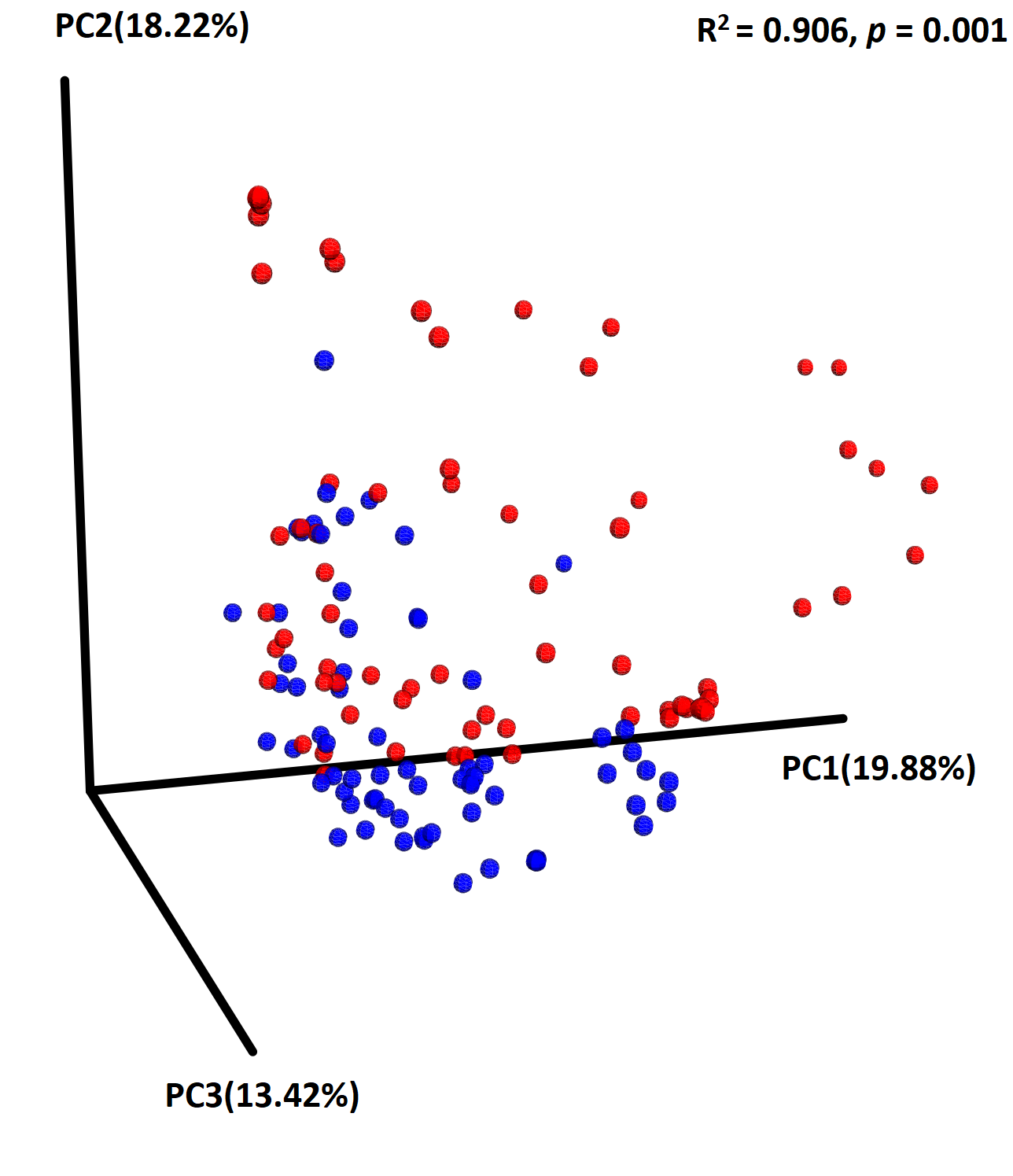

Supplement: Figure S2 — The distance of bacterial communities between human milk (blue dots) and neonatal stool (red dots) samples. The p- value was calculated to compare beta diversities among all samples using ANOSIM (R = 0.198, p = 0.001) and Adonis ( R2 = 0.906, p = 0.001) methods. [file peerj-08-9205-s010.png]

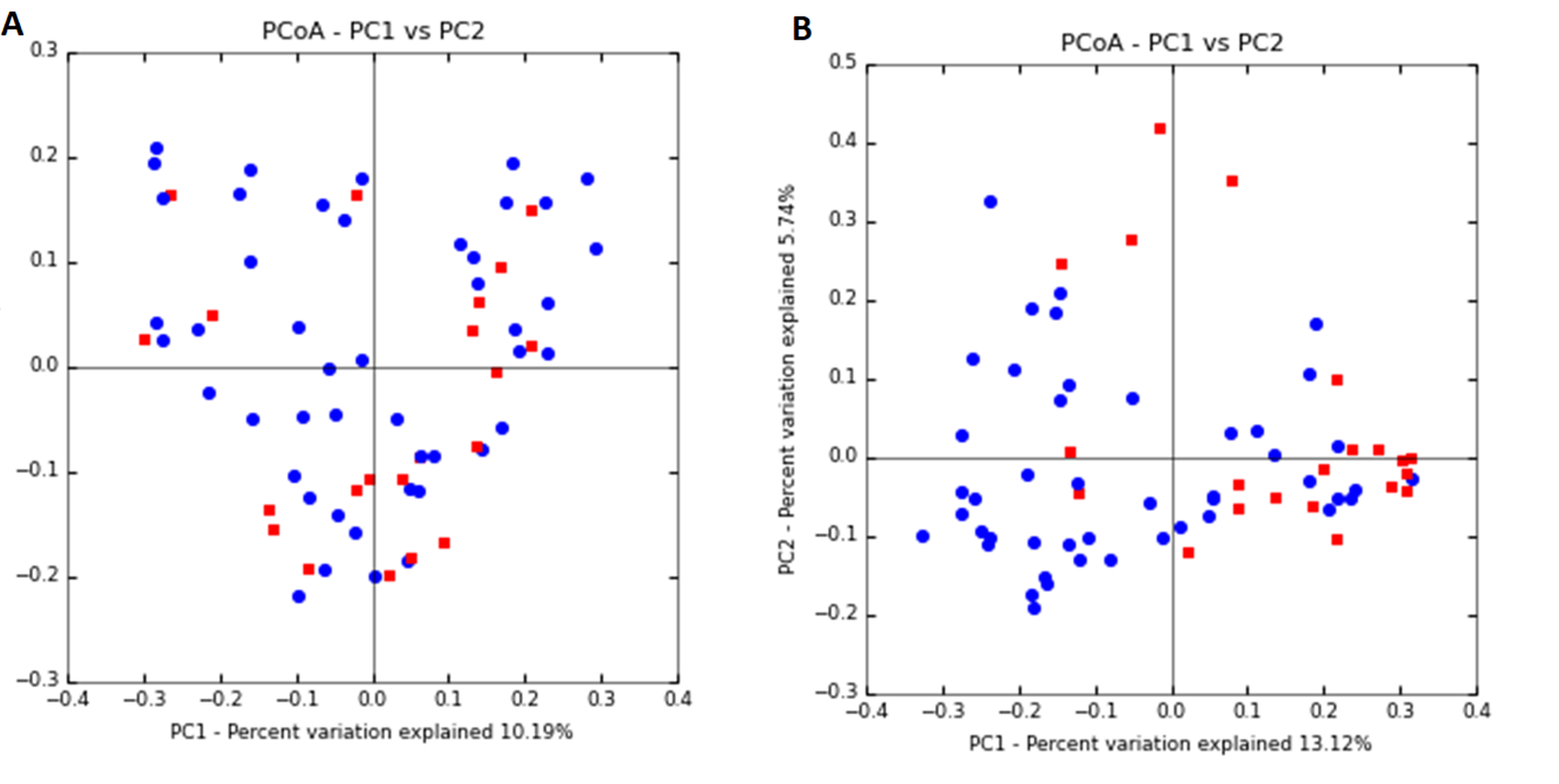

Supplement: Figure S3 — (A) human milk (ANOSIM p = 0.88, Adonis p = 0.71), and (B) neonatal stool (ANOSIM p = 0.006, Adonis p = 0.001) according to the delivery mode. Unweighted UniFrac distances were calculated to evaluate diversity among all samples. Vaginal delivery samples are plotted as blue dots and C-section delivery are represented as red dots. [file peerj-08-9205-s011.png]

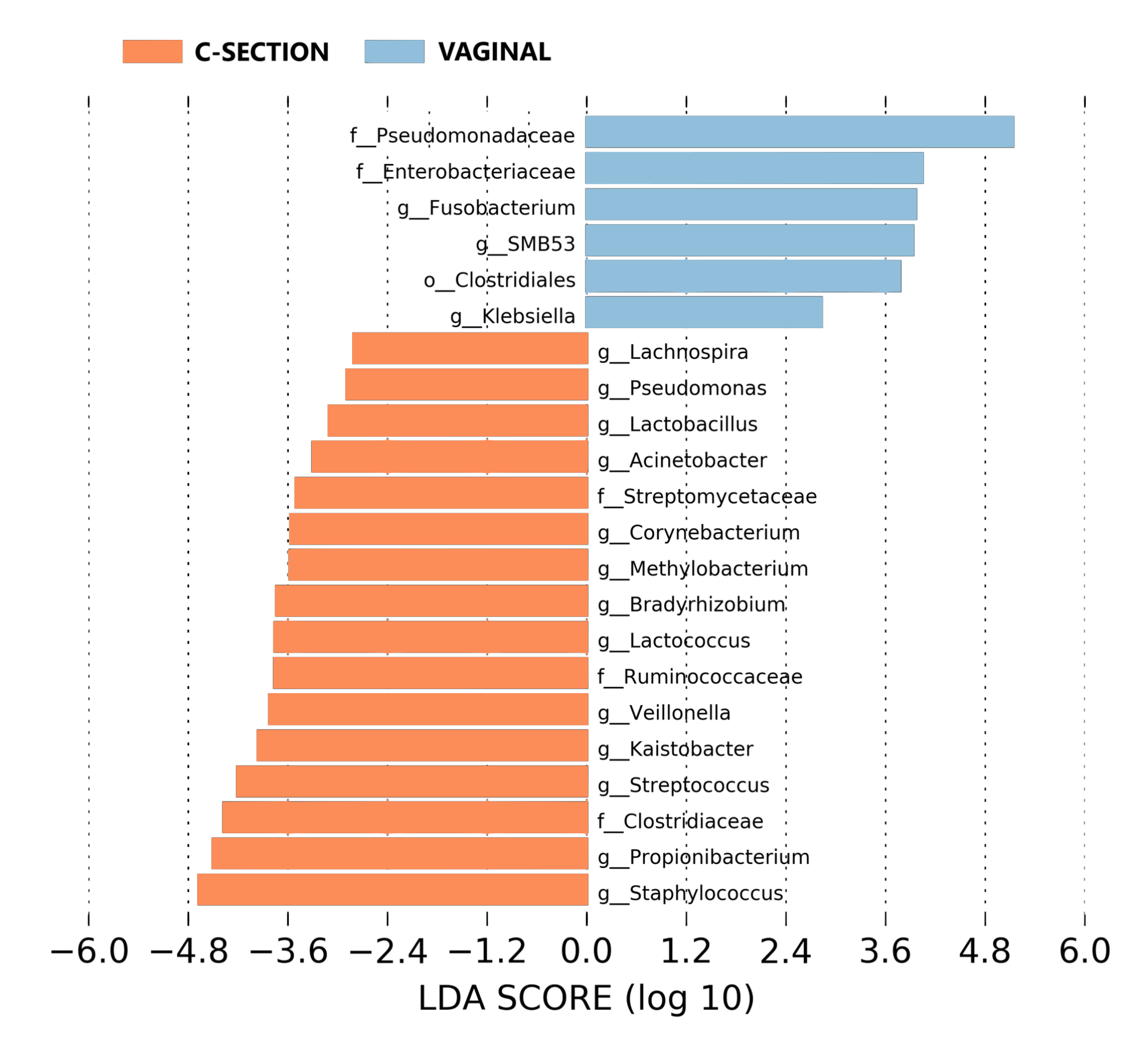

Supplement: Figure S4 — LDA score cutoff of 2.5 was used to discriminate bacterial taxon, p-value < 0.05. Horizontal bars represent the effect size for each taxon: light blue color indicates taxa enriched in vaginal, and crusta color indicates taxa enriched in C-section. [file peerj-08-9205-s012.png]

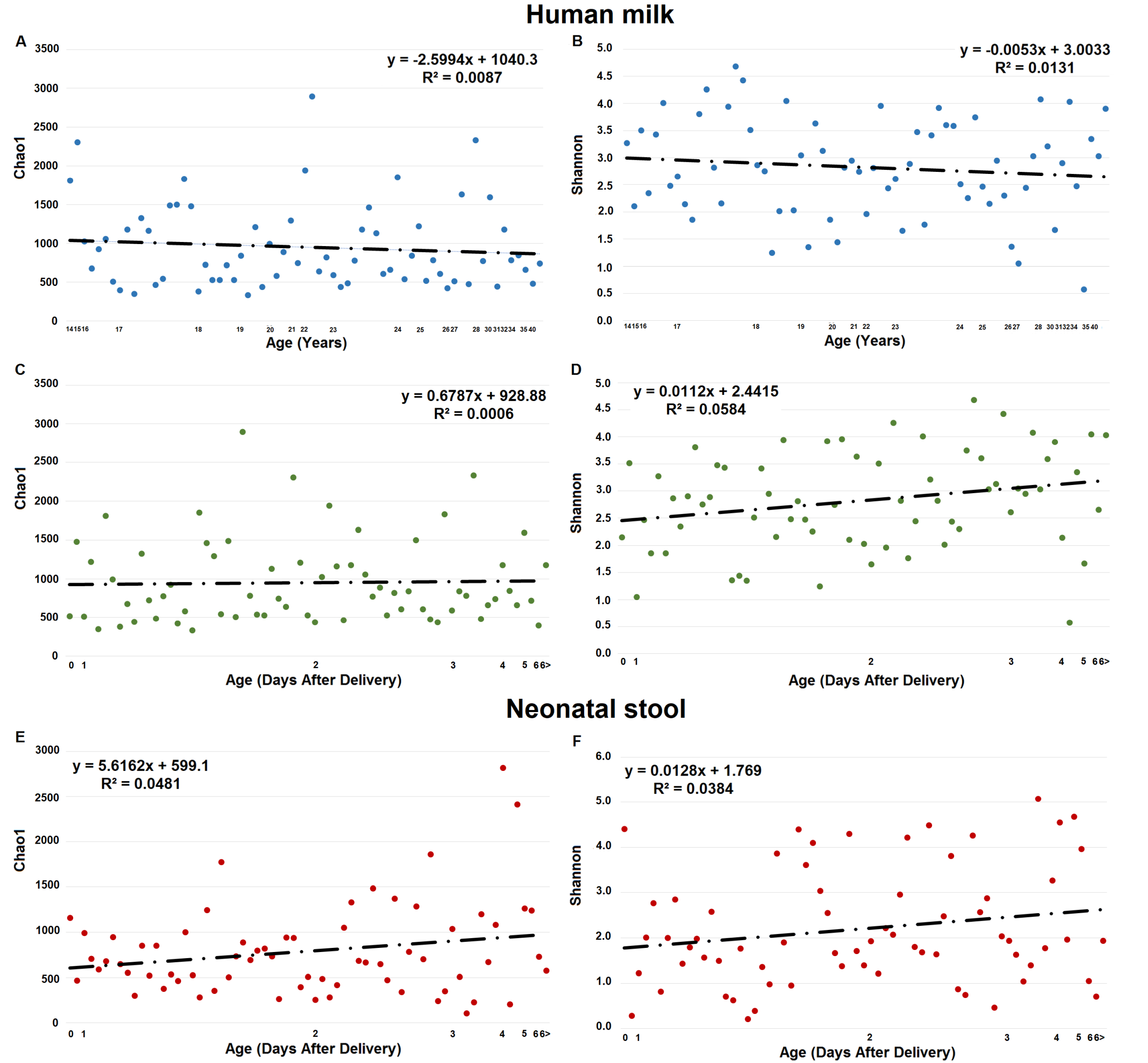

Supplement: Figure S5 — Changes for human milk versus Mother’s age, Chao1 (A) and Shannon (B); Human milk versus days after delivery Chao1 (C) and Shannon (D), Neonatal stool versus Days after delivery, Chao1 (E) and Shannon (F). Indexes are plotted in the Y-axis, and time (years or days) in the X-axis, dots represent individuals, and the stitched lines represent the adjusted curves for the linear regression. [file peerj-08-9205-s013.png]

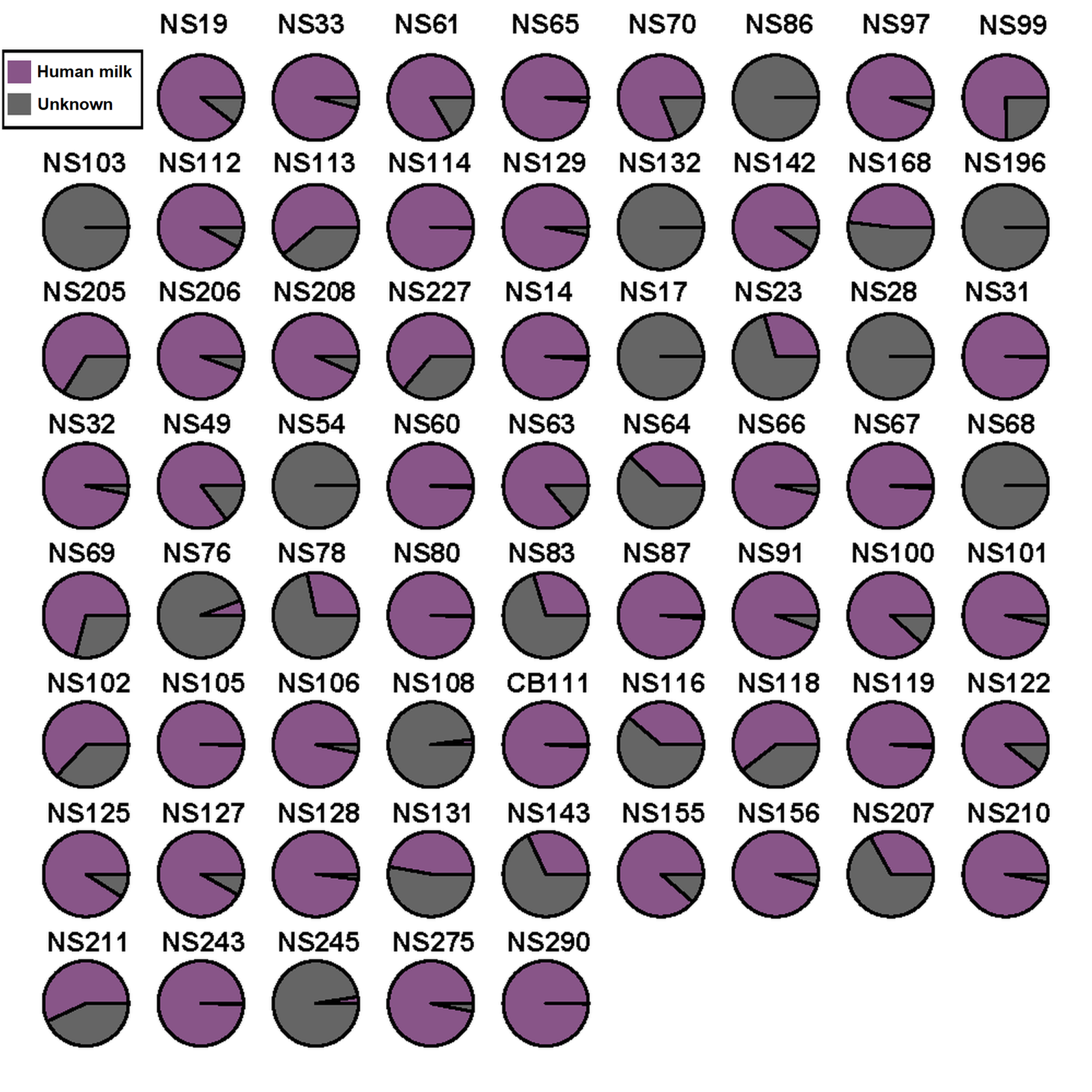

Supplement: Figure S6 — Purple color represents Human Milk origin bacteria, grey color represents bacteria with unknown origin. Numbers above circles indicate the internal code for the sample. [file peerj-08-9205-s014.png]
